# Separability of neural responses to standardised mechanical stimulation of limbs

**DOI:** 10.1038/s41598-017-11349-z

**Published:** 2017-09-11

**Authors:** Emma Brunton, Christoph W. Blau, Kianoush Nazarpour

**Affiliations:** 1 School of Engineering, Newcastle University, Newcastle-upon-Tyne, NE1 7RU Newcastle, UK; 2 Faculty of Medical Sciences, Newcastle University, Newcastle-upon-Tyne, NE1 7RU Newcastle, UK; 3 Institute of Neuroscience, Newcastle University, Newcastle-upon-Tyne, NE2 4HH Newcastle, UK

## Abstract

Considerable scientific and technological efforts are currently being made towards the development of neural prostheses. Understanding how the peripheral nervous system responds to electro-mechanical stimulation of the limb, will help to inform the design of prostheses that can restore function or accelerate recovery from injury to the sensory motor system. However, due to differences in experimental protocols, it is difficult, if not impossible, to make meaningful comparisons between different peripheral nerve interfaces. Therefore, we developed a low-cost electronic system to standardise the mechanical stimulation of a rat’s hindpaw. Three types of mechanical stimulations, namely, proprioception, touch and nociception were delivered to the limb and the electroneurogram signals were recorded simultaneously from the sciatic nerve with a 16-contact cuff electrode. For the first time, results indicate separability of neural responses according to stimulus type as well as intensity. Statistical analysis reveal that cuff contacts placed circumferentially, rather than longitudinally, are more likely to lead to higher classification rates. This flexible setup may be readily adapted for systematic comparison of various electrodes and mechanical stimuli in rodents. Hence, we have made its electro-mechanical design and computer programme available online

## Introduction

The development of peripheral nerve prostheses to restore motor and sensory functions in patients with injury to their peripheral nervous system is a rapidly growing research area^1–5^. The performance of these prostheses have been shown to be significantly enhanced when sensory feedback is provided to the user^6–9^. To provide sensory feedback, sensory information needs to be reliably recorded and passed onto the user. Sensory information could be obtained from either sensors placed onto the surface of intact or artificial hands^9–15^; or from intact whole nerves in a non-functioning limb^6, 7, 16^. In this paper we deal with the second case where, the subject’s own sensory receptors can be used to provide sensory feedback.

A number of different neural interfaces could be used to record sensory information from peripheral nerves in a non-functioning limb^1, 2^. As such, several studies have already examined recording from peripheral nerves to extract afferent sensory signals using a variety of these interfaces: cuffs^7, 17–20^, split rings^21^, transversal intrafascicular multichannel electrodes (TIME)^22, 23^, longitudinal intrafascicular electrodes (LIFE)^24, 25^, regenerative microchannel electrodes^26^ and Utah electrode arrays (UEA)^27^. Unfortunately, differences in the currently-published experimental protocols render comparisons between these interfaces meaningless, if not impossible. For instance, Raspopovic *et al*.^7^ reported that they standardised the proprioceptive stimulus by ensuring to move the rat’s toes from a horizontal position to about maximum flexion. By comparison Wark *et al*.^27^ reported only that they measured the response to plantar- and dorsi-flexion, but not on the measures taken to standardise this stimuli. In addition, while these studies generally considered three different stimulus types, namely, propriocepiton, nociception, and touch, little effort was made to compare different intensities within a given stimulus type.

Increasing the quality of information that can be recorded from a peripheral nerve will likely increase the information available to the user. Thus, neural interfaces need to be compared in terms of their ability to discriminate sensory signals both in terms of type and intensity. However, uncontrolled variability in the position and intensity of the mechanical stimuli could result in noise in the spatiotemporal representation of neural activity at different levels of sensory pathway, e.g. skin mechanoreceptors as well as neural circuity of the cortex^15, 28^. To circumvent this problem, Oddo *et al*.^15^ used an instrumented fingertip to simulate and deliver approximated touch stimuli to sensory afferents. However, this approach may not be possible for other mechanical stimuli such as proprioception and nociception. Therefore, development of hardware and experimental protocol that that can apply standardised mechanical stimuli (versatile and reproducible with graded intensity) is necessary to enable measurable comparison of different neural interfaces.

To standardise delivery of mechanical stimuli to the rodent limb, recently, a number of commercial apparatus have become available. These include but are not limited to: Rodent pincher-analgesia meter (Bioseb, USA)^29^, MouseMet and RatMet (Topcat Metrology Ltd, UK)^30^, Electronic Von Frey (Bioseb, USA)^31^, HotProd (ToPcat Metrology Ltd, UK)^32^. These devices, however, are expensive and functionally inflexible. As such, both the hardware and the software of these devices would need to be modified to link with other electrophysiolgy devices for neural recording and stimulation. For instance, they do not provide a synchronisation signal and cannot be triggered digitally.

Arduino™-based systems offer a cost-effective alternative for automation and standardisation of stimuli^33–35^. We have developed a low-cost Arduino™-based system to mechanically stimulate the rat limb and provide electric synchronisation signals as an input to a neural recording system. In this paper, we report in detail the developed system and the accompanying MATLAB™ interface. This system can be implemented in other laboratories readily so that meaningful comparisons between different neural interfaces can be made. We show that using this experimental method in addition to basic machine learning classifiers can help to inform the choice of electrode placement on multi-channel cuff electrodes.

In this study, we stimulated the rat hindpaw and recorded from the sciatic nerve. We envision that this method could be used to stimulate any rodent limb, and neural recordings could be made anywhere along the sensory neural pathway. To facilitate the adoption of the proposed system, we have made the electro-mechanical design of this system and the accompanying MATLAB™-based graphical user interface available online.

## Methods

The objective of this work is to provide a standardised method for comparing different neural interfaces for recording of sensory information for use in closed-loop neural prostheses. The recorded neural signals can be analysed offline to determine possible methods for discriminating stimulus type and strength. The results from these studies will aid in the design of neural interfaces for recording and discriminating sensory information.

### Electronic Design

**Figure 1:**
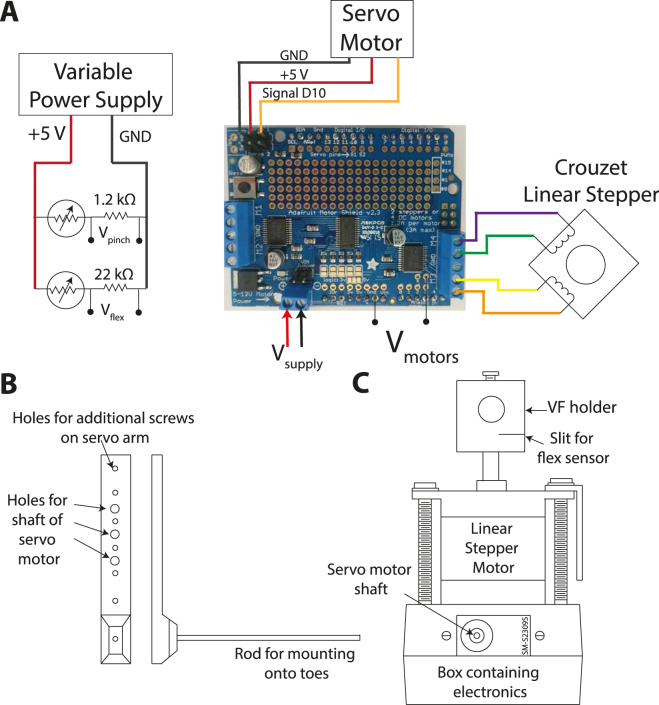
Design of the electro-mechanical hardware for applying the proprioceptive, touch and nociceptive stimulus. (**A**) A simplified circuit diagram showing the pressure and flex sensors connected to the variable power supply, in addition to the motor connections to the Adafruit™ Motor Shield board. The Adafruit™ motor shield board was mounted onto the Arduino™ using standard headers. (**B**) The rod that was attached to the shaft of the stepper motor for proprioceptive stimuli. (**C**) The stepper motor was mounted on top of the box housing the Arduino™, and a piece of plastic was mounted to the end of the stepper motor to hold a Von Frey fibre for touch stimulation. A slit was cut into the side of the mount to hold the flex sensor.

To standardise the intensity of the stimuli across different trials, a system was built using the Arduino Uno Rev3 and Adafruit™ motor shield board (v2.3). The Adafruit™ board was stacked onto the Arduino™ using standard headers. The system was designed to ensure that all three stimulus types, namely, proprioception, touch and nociception, were of a measurable intensity, and a synchronisation signal was provided through a BNC connector to indicate beginning and cessation of stimulus application. An overview of the electrical connections is shown in Fig. 1A. The physical design of the system is shown in Fig. 1(B and C).

The sub-system delivering proprioceptive feedback consisted of an aluminium lever arm with a protruding rod that was attached to a small servo motor (SM-S2309S) (Fig. 1B). The motor was connected to servo port 1 on the Adafruit™ motor shield (Fig. 1A). To provide a synchronisation signal a flex sensitive resistor was connected in series with a 22 kΩ resistor and powered separately by a variable power supply. The voltage was measured across the series resistor via a BNC connector.

The touch sub-system consisted of a Von Frey (VF) fibre holder mounted onto a linear stepper motor (Crouzet 43 N Electric Linear Actuator) (Fig. 1C). A slit was cut in the VF holder so that the flex sensor could be attached to provide an analogue synchronisation signal (Fig. 1C). The linear stepper motor was connected to M3 and M4 pins on the Adafruit™ motor board (Fig. 1A). Since the stepper motor draws a large current, the Arduino power cannot be used to power it and an additional power supply is needed. We used six AA batteries, but a portable power supply may also be used. The control signal to both the stepper and servo motors was also monitored through a BNC connector.

The nociception sub-system comprised a force sensitive resistor (FSR) mounted onto insulated forceps. The voltage across a 1.2 kΩ resistor connected in series with the FSR was measured to provide information about the strength of the stimuli and also provide a synchronisation signal (Fig. 1A).

### User Interface

A graphical user interface (GUI) was developed in the MATLAB^TM^ environment (version 8.5.0/197613 (R2015a); The Mathworks Inc.) to control application of the stimuli, addition of comments to the recording file and file storage. To communicate with the Microcontroller and Neural Signal Processor (Blackrock Microsystems), the support package for Arduino™ and the CBMEX MATLAB extension (Blackrock Microsystems) were used, respectively.

### Animal preparation

All animal care and procedures were performed under appropriate licences issued by the UK Home office under the Animals (Scientific Procedures) Act (1986) and were approved by the Animal Welfare and Ethical Review Board of Newcastle University. Three Sprague Dawley rats weighing 350–450 g were used in this study. Animals were housed under a 12 h light/dark cycle with ad libitum access to food and water.

The animal was initially anaesthetised by an intraperitoneal injection of medazolam and fentanyl/fluanisone (hypnorm) (1:1:2, hypnorm:medazolam:water) with an initial does of 2.7 mL/kg^36^. The animal was then placed onto heating pad with feedback probe to monitor surface temperature. Oxygen was delivered through a nose cone. A rectal thermometer was used to monitor internal temperature, and a pulse oximeter was used to monitor oxygen saturation and heart rate. A subcutaneous injection of meloxicam (1 mg/kg) was given to help maintain anaesthetic depth and fluids were maintained through a tail vein cannula with 0.05 mL of KCl, 10 mL of 0.18% saline 8% glucose and 10 mL water delivered at 2 mL per hour. Anaesthetic depth was maintained via isoflurane in oxygen delivered through the nose cone and additional intraperitoneal injections of the midazolam and hypnorm cocktail. During recordings of sensory signals maintenance doses of the cocktail were given so as to keep the Isoflurane level below 0.5% as Isoflurane has been shown to interfere with neural signal propagation at high doses^37^. Adequate anaesthetic depth was indicated by absence of withdrawal in response to a toe pinch, and the presence of a regular respiratory rhythm. At the end of the experiment, the animal was humanely killed with an overdose of pentobarbitol without recovering from the anaesthesia.

### Surgery

**Figure 2:**
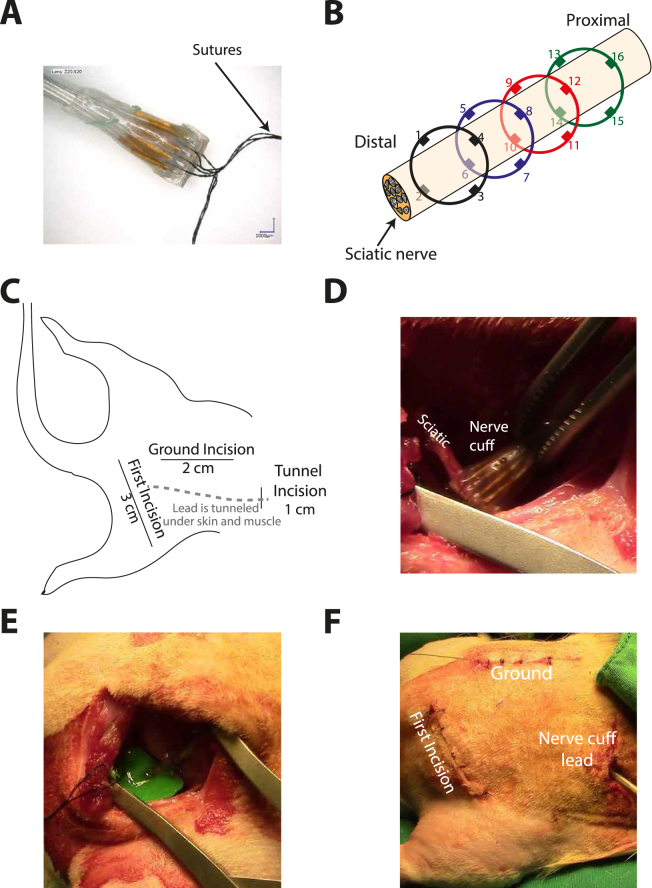
Overview of the surgical procedure. (**A**) Microscopic image of the used 16-contact nerve cuff; (**B**) Relative position of the electrode contacts and the sciatic nerve; (**C**) Illustration of the incisions made, and the nerve cuff lead tunnelled under the skin; (**D**) The 16-contact cuff implanted around the sciatic proximal to where the nerve branches into its peroneal, tibialis, and sural components; (**E**) Use of Kwik-Cast to secure the cuff in place; (**F**) The muscle and skin closed in preparation for the animal to be moved onto the sling.

An overview of the surgical protocol is shown in Fig. 2. A 16-contact cuff (Microprobes for Life Science, Gaithersburg, USA, Fig. 2A) was implanted around the sciatic nerve on the right hindpaw. Each cuff had 16 platinum wire electrode contacts (four rings of four electrode contacts, Fig. 2B) mounted on a silicon rubber tubing. The inner diameter of the cuff was 1 mm. The rings were spaced 0.75 mm apart and the distance from the end of the contacts to the end of the cuff was 1 mm. Sutures placed within the cuff were used to aid handling.

An incision of about 3 cm was made in the skin beginning in line with the spine approximately 1 cm posterior from where the femur attaches to the hip and finishing about 1 cm from the knee. From this point onwards the tissue was regularly wet with saline to prevent drying out. The two planes of the biceps femoris were then blunt dissected to expose the sciatic nerve and the sciatic nerve was carefully freed from the surrounding tissue.

An incision of about 1 cm was made approximately 3 cm rostral to the first incision. A tunnel was made under the skin and muscles between the two incision sites using blunt dissection (Fig. 2C). The nerve cuff was threaded through the tunnel, beginning at the rostral incision. This was performed to bring the nerve cuff closer to the final implantation site and reduce the propensity for compression or stretch of the nerve(Fig. 2D).

The nerve cuff was placed proximal to the point where the nerve branches into its peroneal, sural and tibial components; and positioned without consideration to the electrode contact location in relation to the nerve fibres (Fig. 2D). To secure the cuff in place, Kwik-Cast (World Precision Instruments, FL, USA) was placed around the cuff and the nerve (Fig. 2E). The sutures of the cuff were not tied to ease recovery of the nerve cuff for reuse. The muscles and then the skin were then sutured closed to minimise the movement of the cuff and to ensure the tissue did not dry out during the experiment (Fig. 2F). Closing the incision cite and tunnelling the leads of the nerve cuff was performed to reduce movement of the cuff when the animal was moved for the mechanical stimulation.

For the ground electrode, approximately 1 cm of insulation was removed from the end of polytetrafluoroethylene (PTFE) insulated tungsten wire (Advent Research Materials, UK). A 1 cm incision was made in the tissue above the L5 spinous process. The bony spinous process was exposed using blunt dissection and finally a bone scrapper. The ground electrode was then wrapped around the L5 spinous process and secured with dental acrylic. The skin above the spinous process was sutured closed (Fig. 2F).

A stranded stainless steel wire was placed in the skin above the nerve cuff as a reference. A needle was inserted in the skin and the stainless steel wire was threaded through the needle. The needle then was removed leaving the stainless steel wire in place in the skin. The wire was then secured with tissue glue.

### Mechanical Stimulation

**Figure 3:**
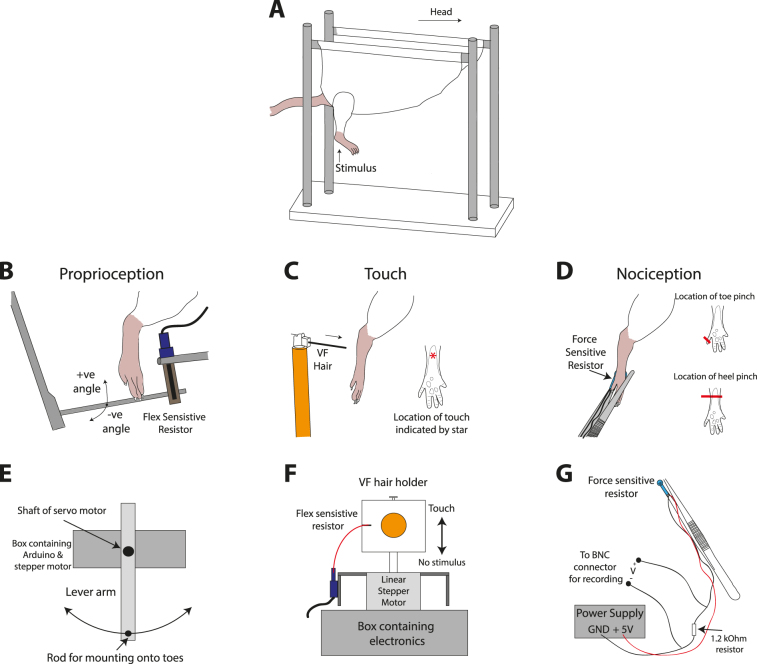
Illustration of experiment procedure. (**A**) Following the surgery, the rat was placed in a sling, allowing for access to manipulate the right hindpaw. (**B**) The dorsum of the nails was glued to a rod so that the foot could be rotated. A positive angle corresponded to plantar-flexion and a negative angle to dorsi-flexion. (**C**) A VF fibre was used to touch the heel of the foot. (**D**) Forceps were used to pinch the foot in two locations the heel and the outer toe (pinky). (**E**) The rod shown in (**A**) was mounted onto a servo motor controlled by an Arduino™. (**F**) The VF fibre was mounted in a holder attached to a linear stepper motor. (**G**) The forceps contained a FSR to measure the applied pressure of the pinch.

At the end of the surgery, the rat was lifted gently and placed into a sling (Lomir Biomedical inc., Canada) so that the right hindpaw was free to move. All other paws remained contained within the sling (Fig. 3A). From this point onwards the Isoflurane was kept below 0.5% and anaesthetic depth was maintained with the intraperitoneal injections of the midazolam/hypnorm cocktail. The three different types of mechanical stimulation were performed sequentially (proprioception, touch and nociception). The order was randomised across the three animals.

#### Proprioception

The dorsum of the rats nails were glued to the bar attached to the lever arm on the server motor (Fig. 3B and E). The flex sensor was placed parallel to the foot on the bar so that movement of the bar, resulted in flex of the sensor (Fig. 3B). The GUI was used to control application of the stimulus and to begin and end file recording.

From a neutral ankle position (~70°), the rats toes were moved to the angle as indicated by the user input into the GUI. For this study six angles were trialled ±10°, ±20° and ±30°, relative to the neutral position. The foot was held at this angle for three seconds before moving back. Initially, a single test trial was implemented to ensure that all angles were within both the foot and the motors range of movement. Each angle movement was repeated 50 times. Holding for three seconds at the desired angle, and then returning to the neutral position for three seconds. The GUI was used to add comments to the file that could later be used for synchronisation.

#### Touch

In this study, two VF fibres corresponding to 100 and 300 grams of force were used. The desired VF fibre was secured in the holder on top of the linear stepper motor (Fig. 3F). The flex sensor was positioned with one end secured to the base of the stepper motor, and the other end placed in a slot connected to the VF fibre holder. When the stepper motor moved forward, the flex sensor would unfold providing a synchronisation signal (Fig. 3F).

The VF fibre was aligned with the area of the hindpaw that was to be tested (Fig. 3C). A scissor stand can be used to move the Arduino box and stepper motor to the correct height. The distance the VF fibre has to move was adjusted with user input to the GUI. Initially, a single test trial was performed using the corresponding pushbutton on the GUI. This was done to ensure that the stepper motor moves the correct distance and touched the hindpaw in the correct position.

The VF fibre was moved by the stepper motor until it touched the foot and bent slightly, held there for three seconds, and then moved back to its starting position. The next trial began after three seconds. We recorded 50 trials for each fibre. Comments automatically added to the recorded data by the GUI could later be used for synchronisation.

#### Nociception

A modified pair of tweezers with a force sensor attached were used to manually pinch the foot. The voltage across the resistor in series with the FSR was measured to provided the synchronisation signal (Fig. 3D and G). The experimenter pinched the foot with the modified forceps holds for approximately one second before releasing for one second. This process was repeated 50 times. The GUI was used to begin and end file recording, and to automatically add comments to the file name so that the size of the VF fibre and position of the touch can easily be identified later.

### Neural recordings

The neural response to the mechanical stimulation was recorded using a 16-contact cuff electrode (Microprobes for Life Science, Gaithersburg, USA). The cuff was connected via an omnetics connector to a digital headstage (Cereplex-M32, Blackrock Microsystems, USA) by which the neural signal was digitised and bandpass filtered (0.3–7.5 kHz). The headstage was connected to the digital hub (Blackrock Microsytems, USA), the signal was converted to an optic-digital format. The signal was then sent to the Cerebus Neural Signal Processor (Blackrock Microsystems, USA) via an optical fibre. The electroneurogram (ENG) signals were recorded at 30,000 samples per second and further digitally bandpass filtered (0.25–5 kHz). The BNC cables measuring the voltages over the resistors in series with the flex sensitive resistor and the FSR were connected to Analogue inputs of the Cerebus system.

### Data analysis and separability

**Figure 4:**
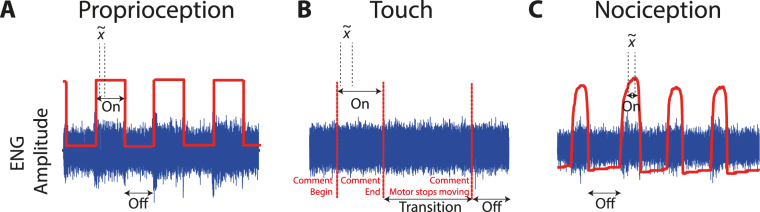
Illustration of the synchronisation signals used to indicate stimulus onset and offset for each stimulus type. This diagram is for illustrative purposes only and is not to scale. The ENG signal is shown in blue. The mean absolute value, $\tilde{x}$, was calculated over 500 ms beginning 250 ms after stimulus onset was detected for all three conditions: (**A**) Onset and offset of proprioception was indicated by the rising and falling edge of the signal recorded from the flex sensor (red). (**B**) Onset and offset of the touch stimuli was indicated by comments added automatically to the neural recording. The transition period indicates when the stepper motor was moving and we do not know if the VF fibre was in contact with the foot or not. (**C**) Onset and offset of the nociception stimuli was indicated by detecting the rising and falling edge from the pressure sensor.

The neural recordings were classified offline for the stimulus type. The signals were first bandpassed filtered in MATLAB using a finite impulse response filter of order 50 (0.8–2.2 kHz). Detection of stimulus onset and offset times was stimulus-specific. For proprioception, the signal recorded from the flex sensor was used. For touch, comments automatically added to the neural recordings by the GUI were used. Finally, for nociception the signal recorded from the pressure sensor was used (Fig. 4).

In all cases, the mean absolute value (MAV) of the neural data from each of the 16 channels was calculated over a 500 ms window beginning 250 ms after stimulus onset was detected. These values were then normalised by subtracting the MAV calculated during stimulus off times. The MAVs for each animal (50 per stimulus type) were then used as the input for the classifier. The MAV was calculated over a period when the motors were not moving. This ensured neural data were not contaminated with artefacts caused by motor movement.

The signal to noise ratio (SNR) for each stimulus type was calculated as in Raspopovic *et al*.^7^. The SNR was given as the ratio between the mean MAV of the ENG signals recorded during stimulus “on” time and the mean MAV recorded during the stimulus “off” time for the sixteen electrodes;

1 $$SNR=\frac{mean({{\rm{MAV}}}_{stimulus\_on})}{mean({{\rm{MAV}}}_{stimulus\_off})}.$$

A quadratic support vector machine was implemented in MATLAB™ (Mathworks inc., ver. 8.5.0.197613, USA) and used for classification. Ten stimulus classes were considered: ±10°, ±20° and ±30°, touch with 100/300 g fibres, heel and pinky pinches. The data was partitioned into five folds and the correct classification rate (CCR) was calculated after cross-validation. We only considered the steady-state response of the nerve, and hence the ENG signals recorded during the transition periods were not classified. Previously, we reported preliminary results on the separability of data collected using this method within stimulus type^38^. In this paper, we examine this in more detail and investigate the separability of data when all ten classes are considered.

For visualisation of separability, the normalised MAVs recorded from each stimulus type were scatter-plotted on the same graph for each animal, that is, the MAV recorded from one electrode was on the *x*-axis, and from a different electrode on the *y*-axis. To further investigate the number and distribution of electrodes that may be required to discriminate between different neural signals, we ran a sequential forward search^39^. With this analysis we aimed to find a subset of electrodes that provided the highest CCR.

**Figure 5:**
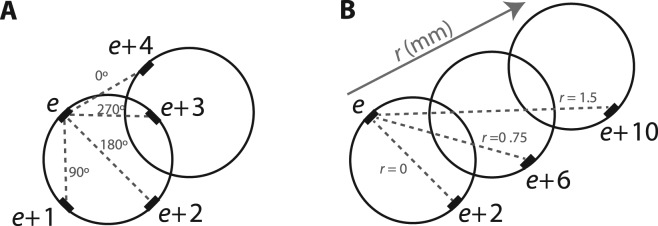
Statistical analysis: (**A**) Description of four different electrode positions circumferentially placed around the nerve for pairwise comparisons; (**B**) Description of three different electrode positions longitudinally placed along the nerve for pairwise comparisons.

In addition, in the case where only two electrodes were used, we asked what the best position of these two electrodes would be. We considered circumferential and longitudinal positions, separately. Circumferentially for each electrode, a second electrode could be placed in one of four different circumferential locations (Fig. 5A). The electrode could be placed along the same side of the nerve but more proximal, e.g. electrode *e* and *e* + 4 with 0° circumferential separation; on the opposite side of cuff, e.g. electrode *e* and *e* + 2 with 180° circumferential separation; adjacent and separated by 90°, e.g. electrode *e* and *e* + 1; or adjacent and separated by 270°, e.g. electrode *e* and *e* + 3. Longitudinally, for each electrode, a second electrode could be placed on the opposite side of the nerve, but on a different ring to the first (Fig. 5B).

For both the circumferential and longitudinal cases, we examined the improvements Δ made to the CCR by the addition of a second electrode in one of the different positions either circumferentially or longitudinally;

2 $${\rm{\Delta }}=\frac{{{\rm{CCR}}}_{e,e+i}-{{\rm{CCR}}}_{e}}{{{\rm{CCR}}}_{e}}\times 100.$$

For the circumferential case, we chose not to consider electrodes 13–16 (on the most proximal ring), since appropriate pairwise comparisons could not be made with the 0° position. For the longitudinal case, we chose not to consider improvements made to electrodes on the last two rings 9–16 so that appropriate pairwise comparisons could be made.

### Statistical analysis

To compare the classification performance using information from a different number of electrodes within animals, that is changing the length of the feature vector used for classification, an asymptotic McNemar’s test was used and implemented in MATLAB™ (Mathworks inc., ver. 8.5.0.197613, USA). To make this comparison each classifier was trained with half of the data and then tested with the other half giving *n* = 250. The testing and training sets were kept the same for each classifier to make an accurate comparison. Within each animal a pairwise McNemar’s test was conducted between the classifier using *e* and *e* + 1 electrodes (*n* = 250), 15 paired-tests were performed within each animal. A significance level of 5% was used. Pairwise significance values were corrected using the Bonferroni correction for multiple comparisons. As the test was repeated 15 times within each animal, a *p* value less than 0.003 indicated significance.

To compare the classification rates of different combinations of electrode pairs in terms of their circumferential or longitudinal separation, statistical analysis was performed in SPSS (Ver. 23, IBM Corporation, USA). A non-parametric independent samples Kruskal Wallis Test was used to measure significance between electrodes being placed at different positions circumferentially (*n* = 12) or longitudinally (*n* = 8) around the nerve as some of the samples defied normality according to the Jarque-Bera test. Pairwise significance values were corrected using the Bonferroni correction for multiple tests. In the circumferential case the pairwise test was repeated 6 times in the same dataset thus a *p* value less than 0.008 was considered to be significant. In the longitudinal case the pairwise test was repeated three times thus a *p* value less than 0.016 was considered significant.

### Data availability

To facilitate the adoption of the proposed system, we have made the electro-mechanical design of this system and the accompanying MATLAB^TM^-based graphical user interface available online. Data supporting this publication is openly available under an ‘Open Data Commons Open Database License’. Additional metadata are available at: http://dx.doi.org/10.17634/141353-2. Please contact Newcastle Research Data Service at rdm@ncl.ac.uk for access instructions.

## Results

The ENG signals were recorded in response to sensory stimulation of the hindpaw. The largest SNR occurred at the most extreme proprioceptive angles ±30° and when a pinch of the heel was performed. By comparison, the smallest SNR occurred when either of the touch stimuli were applied. For both of the touch stimuli, in all animals the SNR was close to 1.

The MAV feature of the recorded data from all 16 electrodes contacts was extracted and used as input to a classifier. Table 1 shows the mean SNR for all of the different stimuli presented. Table 2 lists the CCR for the different stimuli individually, as well as combined for each animal. In all three animals the touch stimuli had the lowest CCR, whereas much larger CCRs were seen for proprioception and nociception.

**Table 1:** Mean ± the standard deviation of the signal to noise ratio recorded on the 16 electrodes for each of the different stimuli applied.

	Proprioception						Touch		Nociception	
	−30°	−20°	−10°	10°	20°	30°	100 grams	300 grams	Pinky	Heel
Animal 1	1.36 ± 0.07	1.32 ± 0.07	1.19 ± 0.04	1.08 ± 0.03	1.30 ± 0.06	1.48 ± 0.09	1.08 ± 0.01	1.15 ± 0.02	1.18 ± 0.03	1.38 ± 0.05
Animal 2	1.21 ± 0.05	1.17 ± 0.04	1.01 ± 0.02	1.14 ± 0.03	1.25 ± 0.05	1.38 ± 0.07	1.04 ± 0.01	1.03 ± 0.00	1.19 ± 0.02	1.33 ± 0.03
Animal 3	1.17 ± 0.03	1.15 ± 0.03	1.02 ± 0.02	1.14 ± 0.03	1.22 ± 0.05	1.49 ± 0.07	1.11 ± 0.00	1.08 ± 0.00	1.08 ± 0.01	1.22 ± 0.01

**Table 2:** Correct classification rate of the different stimuli. The table shows the mean CCR ± the standard deviation of a 5-fold cross validation.

	Proprioception (n = 6)	Touch (n = 2)	Nociception (n = 2)	all (n = 10)
Animal 1	0.99 ± 0.01	0.73 ± 0.08	0.94 ± 0.04	0.83 ± 0.03
Animal 2	0.95 ± 0.03	0.59 ± 0.08	0.80 ± 0.08	0.84 ± 0.03
Animal 3	0.93 ± 0.02	0.63 ± 0.10	0.96 ± 0.02	0.75 ± 0.04

**Figure 6:**
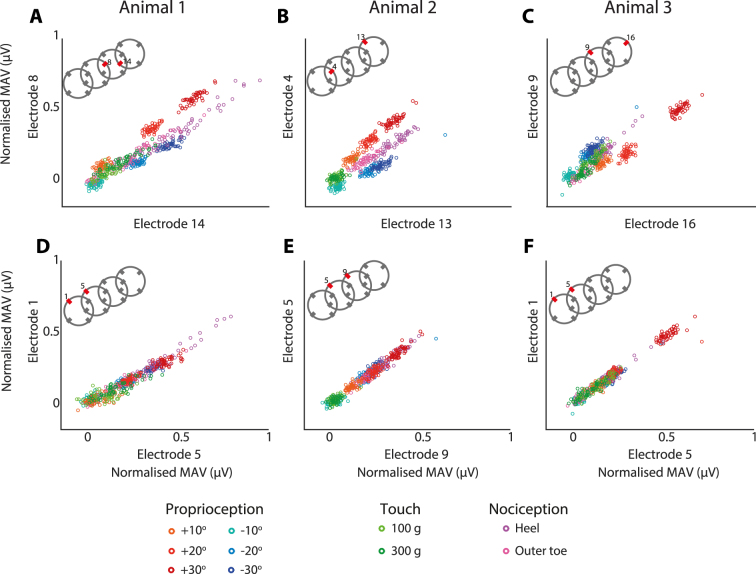
Scatter plots from each animal showing the electrode pairs with the largest correct classification rates (**A**–**C**) and the lowest correct classification rats (**D**–**F**). The *x*-axis is the MAV recorded on a single electrode, and the *y*-axis is the MAV recorded on a single different electrode. The insets of the cuff illustrate the relative positions of the electrodes used in the scatter plots

To further illustrate the separability of the data, the MAVs recorded for selected electrodes were scatter plotted and the CCR calculated considering only two electrodes at a time. Figure 6 shows the scatter plots for all stimulus types for the three animals tested. Figure 6(A–C) show the pair of electrodes in each animal that had the highest CCR and Fig. 6(D–F) show the pair of electrodes in each animal that had the lowest CCR. In all three animals the lowest CCR occurred on electrodes that were placed a single ring apart, on the same side of the nerve. The scatter plots show that when a low classification rate occurs, the data points from each class overlap along a single line extending from the origin of the graph. In comparison, when a high classification rate occurred, the different classes can be seen to lie along different lines. In particular, the positive and negative proprioception responses can be clearly distinguished in separate clusters in the best examples (Fig. 6(A–C)). Whereas in the worst examples, the proprioceptive classes can only be separated in terms of intensity |10°|, |20°| or |30°|, but the clusters indicating the direction of the movement overlap and cannot be separated.

**Figure 7:**
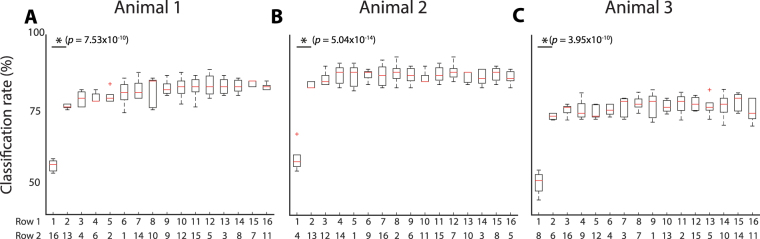
Box plots of the correct classification rate as each additional electrode is added for (**A**) Animal 1, (**B**) Animal 2 and (**C**) Animal 3. Row 1 indicates the number of electrodes (features) used for classification and Row 2 indicates the number of the electrode that was selected. The boxplots show the 5-fold cross validated results. Boxplot description: horizontal red lines, medians; solid boxes, interquartile ranges; whiskers, overall ranges of non-outlier data; red crosses (*red*+), outliers. Asterisks *indicate statistically significant differences. The *p*− values calculated using the McNemar’s test (*n* = 250).

The sequential forward selection algorithm was run to further investigate whether increasing the number of electrodes used as input to the classifier could improve the CCR. Figure 7 shows the classification rate as each additional electrode is selected, with row 1 displaying the number of electrodes used as features. The electrode that was added in each sequential search is shown in row 2. A CCR of a level above chance can be achieved with a single electrode. This can be explained in examination of the scatter plots in Fig. 6, whereby increasing the stimulus intensity results in an increase on MAV on all electrodes, so that a single electrode would be able to separate stimuli of different strengths. Nevertheless, a significant increase (McNemar’s Test, *n* = 250, *p* < 0.003) in the CCR is observed with the addition of a second electrode in all animals. However, including more than two electrodes in the features for the classifier did not significantly improve the CCR (McNemar’s Test, *n* = 250, *p* > 0.003).

**Figure 8:**
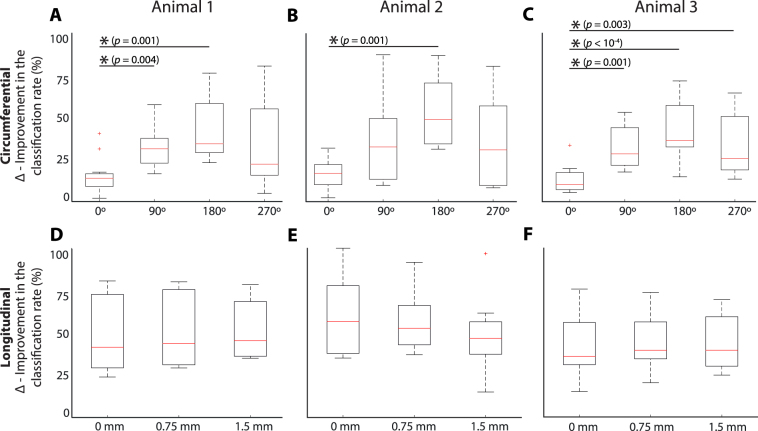
Box plots of the percent improvement to the classification rate by the addition of a second electrode in one of four positions circumferentially (**A**) Animal 1, (**B**) Animal 2, (**C**) Animal 3; or one of three position longitudinally, (**D**) Animal 1, (**E**) Animal 2, (**F**) Animal 3. Boxplot description: horizontal red lines, medians; solid boxes, interquartile ranges; whiskers, overall ranges of non-outlier data; red crosses (*red*+), outliers. Asterisks *indicate statistically significant differences. The *p*-values are calculated with the Kruskal-Wallis test.

Figure 6 shows the choice of the second electrode as input to the classifier is important. Thus, we tested whether we could determine statistically where the second electrode should be placed in relation to the first to increase the likelihood that a higher CCR would be achieved. We considered this separately for circumferential and longitudinal separation. Circumferentially for each electrode, a second electrode could be placed in four different circumferential locations on one of the rings in relation to the first: 0°, 90° 180° or 270° (Fig. 5). For electrodes 1–12 we examined the improvements made to the classification rates by addition of a second electrode in one of the four listed locations. Figure 8(A–C) show the resultant box plots for circumferential separation. In all three animals electrodes placed 180° apart have the largest median improvement to the CCR and electrodes placed at 0° the lowest median improvement. Additionally in all three animals electrodes placed at 180° showed a significantly larger improvement to the CCR than electrodes placed at 0° (Kruskal-Wallis, *n* = 12, *p* < 0.008).

Longitudinally for each electrode, a second electrode could be placed opposite but on one of three different rings separated by 0, 0.75 or 1.5 mm. For electrodes 1–8, we examined the classification rates by addition of a second electrodes in one of the three listed locations. Figure 8(D–F) show the resultant box plots for longitudinal separation. The ring that the second electrode was added to, did not have a significant impact on the improvement to the classification rate in any of the animals (Kruskal-Wallis, *n* = 8, *p* > 0.016). This finding indicates the circumferential position of the electrodes is more important than longitudinal position, in terms of improving the classification rate when a second electrode is added to the ring.

## Discussion

The work presented here is in the context of a recording peripheral nerve prosthesis that has the aim of providing sensory feedback to spinal cord injury or stroke patients who have intact sensory receptors. In recent years, considerable effort has been put into the development of neural interfaces that have the ability to record sensory information from peripheral nerves^2, 8, 9^. This has resulted in a variety of different neural interfaces available to be used in a closed-loop peripheral nerve prosthesis. However, differences in reporting of experimental methods means that it is difficult to make comparisons between neural interfaces that are designed and tested at different laboratories. Here, we have proposed an experimental protocol that will allow for testing to be standardised. The experimental protocol described allows for rodent limbs to be stimulated in a reproducible manner, and for the stimulation applied to be delivered at different measurable intensities. The proposed designed was optimised for when the rat’s hindpaw is mechanically stimulated and neural recordings made from the sciatic nerve. However, the experiment described may be adapted readily for other small animals, and recordings could be made anywhere along the sensory pathway with various neural electrodes.

Being able to synchronise the neural recordings with the stimulus onset and offset is an important part of the design of the experiment. Thus, here we have provided a number of methods for synchronisation. For proprioception and touch three different options for synchronisation were implemented: the voltage over a resistor in series with a flex sensitive resistor that was bent in correspondence to the motors moving was recorded via an analogue input; the control signal to the motors was also recorded via an analogue input; and finally, start and end comments were automatically incorporated in the recording using Blackrock’s NPMK MATLAB library. This provides a variety of options for the experimenter when the data is analysed, or if one of the sensors fail. For the nociception stimulus, only a pressure sensor was placed on the end of the forceps that were used for pinching and this was used to synchronise the neural recordings. In future implementations of this experiment, the pinch could be automated allowing for motor signals and comments to provide information about the beginning and cessation of stimulus application, in addition to the pressure sensor. This would also solve the problem of the pinch stimulus being applied for a shorter period of time.

Three different stimulus types were presented to the rat’s foot (proprioception, touch, and nociception) corresponding to different functional classes of afferent nerve fibres (A*β*, A*α* and A*δ*/C respectively)^7^. It remains difficult to apply a single stimulus type in isolation. For example, in pinching the foot for nociception, the whole foot is touched with the forceps and the foot was seen to move slightly when pinched, thus pinching the foot would also elicit a touch and proprioceptive response inadvertently. Further thought is needed to provide a true nociceptive stimulus. One possible alternative is that a needle could be used to poke the foot and evoke a nociceptive response as Badia *et al*.^23^ explored in their study. Although poking the foot with a needle would result in a reduced touch and proprioceptive contribution to the nociceptive neural recordings, the needle would become blunt with repetitive application and may not provide a reproducible stimulus intensity. Another interesting sensation to study would be the detection of different vibratatory stimuli, although some of this information may already be conveyed to the patient as sound through osseoperception^40^. Nevertheless, the Arduino system would be easy to adapt to apply other stimulus types or intensities depending on the desired purpose of a experiment.

In addition, if this approach would be used to develop a closed-loop functional neural stimulator, a number of improvements to the experimental procedure should be ensured. This includes randomising the order of all trials or interleaving trials of different sensory types with one another to ensure no temporal effects in the data are seen. Keeping the starting position of the leg fixed for all trials, i.e. the leg could remain glued to the bar used for proprioception for both touch and nociception as well, to ensure baseline neural firing rates do not change between trials.

The proposed procedure can be used to study the effects of different electrode configurations on the separability of recorded sensory information. We used the described system to record neural signals in response to mechanical manipulation of the rat’s hindpaw and extracted the MAV feature. Applying a simple classifier to this data allowed us to determine whether neural signals recorded by different electrodes on a multi-channel cuff contain information about both stimulus type and strength. Results showed that, in principle, different types and strengths of sensory information can be classified with the performance of the classifier used above chance in all three animals. Furthermore, we can see from the scatter plots in Fig. 6 that while a single channel is capable of separating information in terms of strength of stimulus, a second electrode is needed to differentiate between stimuli of different types. For example a single channel can show the magnitude of the proprioceptive movement, but not the direction. The addition of a second electrode allows for both the magnitude and direction of the proprioceptive movement to be differentiated. Using only two channels recorded from the sciatic nerve to separate activity from the peroneal and tibial branches has previously shown in a study by Cheng *et al*.^41^. They used a regression model to separate the afferent signals from the tibial and peroneal nerves. In our study we found that the position of the second electrode is important for separation of the different neural signals. Because of the importance of selecting the most informative electrodes, even though the addition of more than two electrodes did not significantly improve classification rates, it would be wise to include a larger number of electrodes so that the most informative electrodes locations around the nerve can be selected. This has also been previously shown by Zariffa *et al*.^42^, where they found increasing the number of electrodes improved the performance of the classifiers used. Their finding was not because of the sheer number of contacts, but because of the opportunity to then select the most informative locations around the nerve.

One limitation is that the cuff used in this study could not be used in chronic experiments while the animal was awake. In awake and moving animals, EMG signals would appear as artefacts on the recording. There are a number of improvements that could be made to the cuff to improve its performance under these conditions. These include: referencing the cuff electrodes in a tripolar fashion^43^, adding gold sheeting to the outside of the cuff^44^ and increasing the distance between the recording electrodes and the edge of the cuff^43, 45^. Despite these shortcomings in the design of the cuff, the CCRs seen are very high, indicating that modifications to the current cuff design could significantly improve CCRs and enable its use in chronic studies. The CCRs achieved in our study are similar to those seen in a study conducted by Raspopvic *et al*.^7^ when only a single channel tripolar cuff was used. However, they saw much larger SNRs than we do in this study. The larger SNR is most likely due to the use of a tripolar reference configuration, whereas, we have referenced all of our electrodes to a wire placed in the skin.

For development of algorithms to be used in real-time applications there are a number of considerations we would need to take into account that we have not included in this study. Firstly, we normalised the features used for classification with signals recorded during stimulus off times, in real-time we would need to include a stimulus “off” class in addition to the 10 stimulus classes. If a stimulus “off” class was included it is likely that the signals with low SNRs would not be separable. This includes the two touch classes, and in some cases the small proprioceptive angles of ±10°. Secondly, we have only examined the steady state or tonic response seen in neural recordings. Nerve recordings also exhibit phasic responses with stimulus application^46^. Addition of data during the onset and offset phase of stimulation will make the problem more complex. The current classification method will most likely overestimate the intensity of the applied stimulus during the initial stimulus application. Finally, in this study we found that if the features extracted were not normalised with stimulus off times, a small shift in baseline noise would alter the MAV enough that the features were classified due to temporal differences in the signal, rather than the stimulus that was applied. A method will need to be implemented to monitor shifts in the baseline noise.

The different proprioceptive classes were highly clustered on the scatter plots in all three animals. This is in agreement with the study by Raspopovic *et al*.^7^, where proprioception was able to be classified from rest with a high CCR. However, our results indicate that a touch stimuli would not be able to be reliably identified from rest. This is in direct contrast to what was seen by Raspopovic *et al*.^7^ and is most likely due to the low SNR seen in this study for the touch stimuli.

Being able to use the MAV feature recorded on electrodes placed circumferentially around the nerve to differentiate between flexion and extension of the foot is perhaps unsurprising. During flexion the extensor muscles are stretched and during extension the flexor muscles are stretched, these are supplied by the tibial and peroneal nerves respectively^18, 46, 47^. At distal locations in the sciatic these branches remain spatially separated, resulting in the relative amplitude of the signal travelling along these branches appearing differently at electrodes placed circumferentially around the nerve. Finite element modelling studies have shown the potential to separate information from different fascicles, using multi-channel cuff electrodes, taking advantage spatial separation of the fascicles in the nerve^48, 49^. In addition, these results are in agreement with previous studies where neural recordings have been made proximal to the nerve branching and stimulation of the nerve branches has been applied^50, 51^. Both of these studies showed that different compound action potential magnitudes were seen at different electrode contacts located on opposite sides of the nerve, depending on the nerve branch that was stimulated. Given this, spatial filters such as fast independent component analysis used by Song *et al*.^18^ are likely to enhance separability of sensory information. Along the same vein, it is perhaps not surprising that increasing the longitudinal distance between two electrodes did not significantly increase the CCR, as the maximum distance we examined here was 1.5 mm. The spatial separation of fibres along the nerve would not be expected to change substantially over this distance. Future work may examine how larger longitudinal distance will affect classification rates.

High CCRs were also seen for the nociception stimuli. This is in contrast to the study by Raspopovic *et al*.^7^ where nociceptive stimuli where found to have the lowest SNRs and to be the most difficult to classify. This was hypothesised to be due to the small size of the fibres that carry these signals and resultant small signal size that are difficult to visualise in whole nerve recordings. In our study a high CCR for nociceptive stimuli is most likely due to activation of many other fibres, due to the large size of the forceps that were used for pinching. Regardless, we have shown that the MAV recorded at different electrodes located circumferentially around the nerve can be used to separate different stimuli.

Currently, there remains a trade-off between specificity of a neural interface and its invasiveness^52^. At first glance one would determine a interface with greater recording specificity would allow for more information to be transferred to the patient; however, current methods of providing sensory feedback to patients provide coarse information about stimulus type and strength^8^ although recent effort show that amputees can perceive graded sensory intensity^19^. Thus, it may be preferred to sacrifice recording specificity to decrease the invasiveness of the implant and reduce the surgical complexity. To choose the best neural interface for the task, there needs to be a robust, easy to implement method for comparison of the different neural interfaces available. This requires a standardised framework. This framework would also need to be capable of measuring the effect size of the improvements different neural interfaces can make. Here, we have made the first efforts to develop such a framework.

The system and experimental protocol described here allow for the rodent limb to be mechanically stimulated with the stimuli applied being of a measurable and reproducible intensity. We have shown that the neural analysis of recordings collected from multi-contact cuff electrodes can be used to guide the development of classification algorithms and the design of neural interfaces for sensory neuroprostheses. We believe that with synergistic experimental and simulation studies, e.g. Kolbl *et al*.^53^ and Koh *et al*.^54^, the suitability of multi-contact cuff electrodes may be further quantified.

Finally, the Arduino™ system can be easily adapted for the experimenters purpose. Ultimately, standardising experimental protocols will allow for neural interfaces being developed at different labs to be compared, resulting in improved selection and function of neural interfaces for a range of specific purposes.
